# RETRACTION: Wu-Tou Decoction Inhibits Chronic Inflammatory Pain in Mice: Participation of TRPV1 and TRPA1 Ion Channels

**DOI:** 10.1155/bmri/9874314

**Published:** 2025-08-07

**Authors:** BioMed Research International

RETRACTION: C. Wang, C. Liu, H. Wan, et al., “Wu-Tou Decoction Inhibits Chronic Inflammatory Pain in Mice: Participation of TRPV1 and TRPA1 Ion Channels”, *BioMed Research International* 2015 (2015): 328707, https://doi.org/10.1155/2015/328707

The above article, published online on 09 March 2015 in Wiley Online Library (http://wileyonlinelibrary.com), has been retracted by agreement between the Editorial Board and John Wiley & Sons Ltd. The retraction has been agreed following an investigation into figure concerns initially raised on PubPeer [1]. Specifically in Figure 5a:

• In the WTD 6.30 g/kg column, the TRPV1 and TRPM8 panels contain unexpectedly repeated elements.
• In the WTD 6.30 g/kg column TRPV1 panel and the control column TRPM8 panel, unexpectedly repeated elements which have been rotated can be seen.
• In the TPRM8 row, the control and CFA panels contain unexpectedly repeated elements which have been rotated.

The authors apologized, explained that this was due to an error in image preparation, and provided revised figures. The response did not satisfy our concerns and it is therefore retracted.

The authors agree with the retraction.
